# Conjunctival Squamous Cell Carcinoma: A Clinical Review of Diagnostic Features, Genetics, Current Management and an Update on Targeted and Immunotherapies

**DOI:** 10.3390/cancers18060940

**Published:** 2026-03-13

**Authors:** Murad Mir, Hardeep Singh Mudhar, Mandeep S. Sagoo, Stephen Gichuhi, Yamini Krishna

**Affiliations:** 1 Department of Eye and Vision Science, Institute of Life Course and Medical Science, University of Liverpool, Liverpool L7 8TX, UK; hlmmir@liverpool.ac.uk; 2 National Specialist Ophthalmic Pathology Service Sheffield, Department of Histopathology, Royal Hallamshire Hospital, Sheffield S10 2JF, UK; hardeep.mudhar@nhs.net; 3 NIHR Biomedical Research Centre for Ophthalmology at Moorfields Eye Hospital, University College London Institute of Ophthalmology, London EC1V 2PD, UK; mandeep.sagoo1@nhs.net; 4 Royal London Hospital, London E1 1FR, UK; 5 Department of Ophthalmology, Faculty of Health Sciences, University of Nairobi, Nairobi 00100, Kenya; sgichuhi@uonbi.ac.ke; 6 Department of Clinical Research, Faculty of Infectious & Tropical Diseases, London School of Hygiene & Tropical Medicine, University of London, London WC1E 7HT, UK; 7 National Specialist Ophthalmic Pathology Service Liverpool, Liverpool Clinical Laboratories, Royal Liverpool University Hospital, Liverpool L7 8YE, UK

**Keywords:** conjunctival squamous cell carcinoma, conjunctival squamous cell carcinoma in situ, conjunctival squamous intraepithelial neoplasia, ocular surface squamous neoplasia, ocular surface carcinoma, ocular oncology, targeted therapy, immune checkpoint inhibitors, immunotherapy, molecular oncology

## Abstract

Invasive conjunctival squamous cell carcinoma (CSCC) is an aggressive, ocular surface malignancy. The mean annual age-standardised incidence rate of 0.45 cases per million per year is increasing with an average annual percent rise of 4.5% and occurs mainly in over 65-year-olds in temperate climates but in a younger demographic in the tropics. Invasive CSCC can lead to vision loss either from the destructive effects of the tumour or side effects of therapy, facial disfigurement from radical surgery, and death from metastases. There is no standardised treatment and not all cases are referred to a specialist ocular oncology centre. Recent progress in cancer immunology and genetics has revolutionised the treatment of cutaneous and head and neck SCCs, which share some similarities to invasive CSCC. A better understanding of invasive CSCC and its preinvasive intraepithelial lesions is required to lead to the development of novel targeted and immunotherapies both for local tumour control, globe sparing alternatives and to prevent disseminated disease. This review aims to provide a comprehensive clinical overview of the current knowledge regarding CSSC, its epidemiology, pathogenesis, presentation, diagnosis, management, recent advances in targeted and immunotherapies for personalised treatment of this disease, and early diagnosis strategies to improve patient outcomes.

## Simple Summary

Conjunctival squamous cell carcinoma (CSCC) is an eye surface cancer that can cause visual loss mortality. It is rare in temperate climates but is common in the tropics, particularly in India and Africa. Global number of new cases is on the rise. There is no standard treatment for CSCC, and management varies between eye cancer centres. This review aims to present current knowledge of CSSC development, clinical presentation, diagnosis, management and outcomes, and finally summarise novel therapies and future directions for research in CSCC. Immune checkpoint inhibitors, particularly anti-PDL1 therapies, offer a less invasive and globe sparing alternative for advanced SCC treatment. Future directions should focus on earlier detection and personalised approaches which integrate immunotherapy and other targeted treatments.

## 1. Introduction

Invasive conjunctival squamous cell carcinoma (CSCC) has a worldwide incidence rate of 0.02 to 3.5 per 100,000, showing increasing incidences in recent decades [1,2,3,4,5]. It typically presents at 60–70 years of age, occurring more commonly in fair-skinned males, of increasing age, and following significant sunlight exposure, but presents at 30–40-year-old females in the tropics [2,4,5,6,7]. It has a high recurrence rate and local spread is common. Distant metastasis occur less commonly [8].

Conjunctival squamous intraepithelial neoplasia (CSIN) [also clinically known as ocular surface squamous neoplasia (OSSN)] is a preinvasive lesion and encompasses a spectrum of intraepithelial squamous dysplasia, from mild to severe, and can progress to in situ squamous carcinoma. Most cases of invasive CSSC develop from CSIN and in situ squamous carcinoma [8,9].

Invasive CSCC usually involves the interpalpebral conjunctiva and limbus (especially the nasal limbus) and can extend onto the cornea. It can also less frequently occur in the caruncle, tarsal, or forniceal conjunctiva. Invasive CSCC presents as a well-demarcated pinkish-grey nodule with keratinisation or ulceration [10]. In the African and Asian population, it often presents with brown surface pigmentation and feeder vessels [11]. It originates from conjunctival epithelium, shows squamous differentiation, and breaches the basement membrane to invade the underlying substantia propria/stroma [6,12,13].

Ultraviolet (UV) radiation and impaired immune surveillance are well known risk factors. Immunosuppression (e.g., due to HIV infection or post-transplant therapy) and oncogenic viruses such as HPV and Epstein–Barr virus (EBV) are important co-factors [2,4,13,14,15,16,17].

Mutations in *TP53*, *TERT*, *EGFR*, *titin*, *HER2* and DNA repair genes and overexpression of matrix metalloproteinases have been identified in invasive CSCC [13,18,19,20,21,22,23,24,25,26,27,28,29].

The treatment for invasive CSCC varies considerably between centres but mainly includes surgical excision and/or adjuvant cryotherapy, topical chemotherapy, brachytherapy, proton beam radiotherapy or external beam radiation. In advanced cases with orbital invasion, orbital exenteration is required [1,6,30,31,32]. Invasive CSCC has a high rate of local recurrence (approx. 10–40%), incidence of up to 24% in regional lymph node metastases and distant metastases of ~6–18% [8,33,34,35,36].

Despite recent successes with targeted and immunotherapies in SCCs elsewhere, data on invasive CSCC treated with similar therapies (EGFR inhibitors, anti-VEGF, anti-PDL1) are promising but limited, often stemming from a single patient or small case series with inoperable or advanced disease prior to surgery [37,38,39,40,41,42,43,44,45,46,47].

This review aims to provide a comprehensive clinical overview of the current understanding of CSCC, its epidemiology, pathogenesis, clinical presentation, diagnosis, and treatments, and explore the recent advances in novel biological therapies and future management.

## 2. Epidemiology

In situ conjunctival squamous carcinoma is the most common preinvasive ocular surface malignancy and if untreated can progress to invasive CSCC. Its incidence varies geographically: in the UK, it is estimated at 0.4 cases/million/year, whereas both Australia and North America show incidences of 19–35 cases/million/year [48,49]. The mean age-standardised incidence rate worldwide is 0.18 and 0.08 cases/year/100,000 among males and females, respectively. However, the highest age-standardised incidence rate reported from Zimbabwe is 3.4 and 3.0 cases/year/100,000 population for males and females, respectively [2].

Invasive CSCC, although rare compared to SCCs of other sites, is a common ocular surface malignancy. It is the most prevalent ocular surface carcinoma representing approximately 4% to 29% of tumours found in the ocular region, depending on the population or tumour registry records [50,51]. There has been a disconcerting increase in incidence in recent decades with markedly advanced cases presenting following the COVID-19 pandemic. Worldwide the incidence is 2–35 per million [1,2,3,4] occurring more commonly in fair-skinned males (with a ratio of 1.83:1 compared with females), of increasing age, and following significant sunlight exposure [4,5,7]. Ultraviolet (UV) radiation is the strongest environmental etiological factor with incidence rate decreasing by 49% per each ten-degree increase in latitude [3].

In the African continent, the disease mainly affects women at a younger age and is strongly associated with human immunodeficiency virus (HIV) infection. It is variable with human papillomavirus (HPV) infection [2,17,52]. The combination of low latitude and high prevalence of HIV and HPV infections most likely explains the higher incidence of invasive CSCC observed in Africa.

A higher prevalence of CSCC is also seen in patients with impaired immune surveillance, certain autoimmune conditions (atopy, ocular cicatricial pemphigoid and xeroderma pigmentosum) and/or immunosuppression with susceptibility to oncogenic viruses HPV and Epstein–Barr Virus (EBV) [2,13,14,15,16]. Iatrogenic immunosuppression is also a risk factor.

## 3. Aetiology and Pathogenesis

The aetiology of CSCC is classically understood to be triggered by UV radiation exposure and immunosuppression (due to HIV infection or post organ transplantation) [4]. Mutations in *TP53*, *TERT*, *EGFR*, *titin*, and DNA repair genes and overexpression of matrix metalloproteinases, Human Epidermal growth factor Receptor 2 (HER2) and programmed death ligand1 (PD-L1) have been identified in invasive CSCC [18,19,20,21,22,23,24,25,26,27,28,29]. However, none of these are pathognomonic, and have been reported in other conjunctival epithelial neoplasms, e.g., pterygia and pingueculae.

The vulnerability of limbal stem cells to the oncogenic effects of UV radiation and certain viruses is postulated to give rise to most invasive CSCC [13]. UVB exposure may induce preinvasive disease by an increase in pyrimidine dimers, which damage DNA and disrupt its repair pathways. Proliferation of unrepaired DNA by continued excess UVB exposure or immunosuppression can manifest CSIN/in situ carcinoma (preinvasive disease) of the conjunctiva [21,53]. UV exposure also activates c-Jun N-terminal kinase 1 (JNK1), which plays a role in tumour development through phosphorylation and oncogenic signal transduction pathways [54].

UVB radiation has also been implicated in upregulated expression of matrix metalloproteinases (MMPs) (particularly MMP-9 and -11) and tissue inhibitors of metalloproteinases (TIMPs) such as TIMP-2 and -3, which promote the tumourigenesis via antiapoptotic, proliferative, invasive and metastatic properties [21].

UV-mediated driver mutations in *telomerase reverse transcriptase* (*TERT*) *promoter* and *TP53* have been reported in up to 48% of in situ and invasive CSCC cases. Telomerase overexpression in the conjunctival epithelium increases telomere length with proliferative and anti-apoptotic functions [20,55]. *TP53* mutations disrupt pyrimidine dimer repair pathways [53]. *TP53* mutations were the most frequent, followed by *CDKN2A* and *PIK3CA* [28]. The role of *stratifin* acting as a p53-mediated inhibitor is also implicated in UV-induced p53 pathogenesis [56].

*Titin* and *EGFR* mutations have been reported in both in situ and invasive CSCC. Structural disturbances in nuclear proteins and EGFR translocation from the membrane into the cytoplasm, respectively, are thought to facilitate oncogenic activity [18]. The cytoplasmic staining of EGFR compared with membranous staining has been shown to be an inverse prognostic marker with increased number of orbital exenteration cases [19].

Overexpression of HER2 has been documented in high-grade CSIN and invasive CSCC suggesting its main role in tumour progression rather than early oncogenic initiation. Anti-HER2 therapy may therefore be a considered in select patients with advanced disease when conventional topical or surgical treatments are contraindicated [24]. However, there are no clinical trials investigating the role of anti-HER2 treatment on invasive CSCC.

Increased vascular endothelial growth factor (VEGF) and alpha-B crystallin protein expression has also been shown in invasive CSCC. VEGF is an established potent angiogenic factor and alpha-B crystallin is known to promote angiogenesis as a molecular chaperone of VEGF. Furthermore, treatment of invasive CSCC with topical mitomycin C reduced the expression of both [57].

PD-L1 is expressed in around 47% of CSSC, with higher expression levels correlating with invasive and advanced stage (≥T3) tumours with increased densities of tumour-infiltrating lymphocytes [58,59]. These results are encouraging and may potentially mirror some success in a subset of invasive CSCC as established targeted therapies and PD-L1 targeted treatment blockades used in head and neck and cutaneous SCC.

Since the 1990s, increased incidence rate of invasive CSCC in Africa has been strongly associated with the high prevalence of HIV primarily as well as EBV and HPV (particularly HPV-16 and -18 subtypes, and less commonly HPV-5 and -8) in patients with HIV. HIV has an immunosuppressive effect on the limbal stem cells, making them vulnerable to oncogenic viruses and leading to aggressive invasive CSCC at a younger age with worse prognosis [60]. HPV-driven invasive CSCC in immunocompetent patients has been controversial but a recent meta-analysis concluded an eight-fold increase in development of CSIN or invasive CSCC in HPV-infected patients compared to healthy control subjects [61]. HPV-16 with early gene expression (active HPV mRNA transcripts confirmed via RNAscope) has been implicated in in situ carcinoma in a subset of patients who had atopy [62]. Comparison of these HPV-positive tumours with HPV negative tumours showed that HPV-positive tumours were very often located in an inferomedial location under the lower eyelid, were not leukoplakic and had higher recurrence rates and were resistant to topical chemotherapy, when compared to HPV negative tumours. Another study further confirmed that patients with HPV-positive tumours were significantly younger and had a higher recurrence rate compared to patients with HPV-negative tumours. The HPV-positive tumours showed a non-keratinising histomorphology [63].

**Figure 1:**
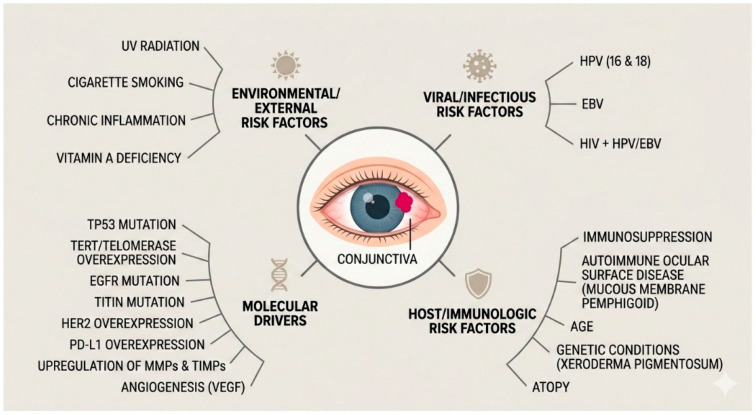
Infographic summarising the aetiology and pathogenesis of CSIN and invasive CSCC (generated in Google Gemini3 Flash).

Other risk factors include cigarette smoking, chronic inflammation and vitamin A-deficiency. Also there is an increased risk of CSIN/invasive CSCC with autoimmune ocular surface disorders such as ocular cicatricial pemphigoid/mucous membrane pemphigoid, and genetic conditions including and xeroderma pigmentosum [2,14,15]. Please see Figure 1.

## 4. Clinical Presentation and Assessment

CSIN and invasive CSCC most commonly occur in the interpalpebral conjunctiva and limbus (usually nasal limbus). Invasive CSCC frequently invades into the cornea. The caruncle, tarsal, or forniceal conjunctiva are less frequently involved. Advanced or recurrent invasive CSCC may extend to the tarsal and forniceal conjunctiva. The latter locations are also seen in immunosuppressed/immunocompromised patients and in those with atopy-associated squamous carcinoma [62].

**Figure 2:**
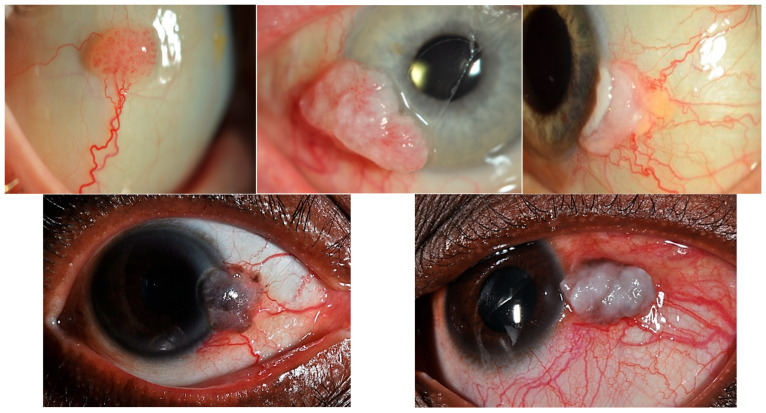
Anterior segment photographs of invasive CSCC with presence of feeder vessels. Middle top row and bottom row images show corneal invasion. Brown surface pigmentation is seen in patients from the tropics (image bottom right). Images on right (top and bottom) shows surface keratinisation.

Most CSIN lesions appear as unilateral, gelatinous and are minimally elevated but can also be papilliform or a leukoplakic plaque (due to keratinisation). However, intraepithelial lesions can be clinically indistinguishable from the invasive disease and hence histopathological diagnosis remains the ‘gold’ standard. Invasive CSCC usually presents as a unilateral, elevated, immobile, well-demarcated pearly pink-to-grey nodule, which can be ulcerated, leukoplakic, gelatinous, or papilliform. They often have feeder blood vessels and intrinsic vasculature (Figure 2). Pigmentation is variable, depending on the Fitzpatrick skin type. Patients may present on noticing a mass with/without pigmentation on their eye but can also have significant visual morbidities, such as irritation/burning with redness and reflex tearing, dry eye, pain, vision disturbance, double vision or even vision loss [35,36,64,65,66,67].

Differential diagnoses of invasive CSCC, include sebaceous cell carcinoma, naevus, melanoma, lymphoma, or eyelid basal cell or squamous cell carcinoma invading the conjunctiva. CSIN can develop in pinguecula, pterygium, viral squamous papillomas and other inflammatory conjunctival conditions. Diagnosis is confirmed on histopathological assessment. Histological assessment in a specialist centre regularly reporting ophthalmic specimens is recommended for accurate diagnosis and grading [8,50,68].

Clinical examination involves slit lamp biomicroscopy and regular colour photograph-documentation of the anterior segment (including with eversion of eyelids). Toluidine blue (0.05%) vital stain has been shown to be a good screening tool for OSSN/CSIN and can assist in identifying the tumour borders, but is not in widespread use due to high false positive rates [69]. Anterior segment optical coherence tomography (AS-OCT) has been used to distinguish OSSN from other conjunctival lesions. In OSSN/CSIN, the AS-OCT features are those of a hyperreflective lesion that has thickened epithelium, and an abrupt transition between normal and abnormal tissue [70]. However, squamous metaplasia is indistinguishable from OSSN on AS-OCT, highlighting the need for biopsy and histopathological analysis of lesions especially where there is overlap [71]. Other modalities such as ultrasound biomicroscopy and confocal microscopy can be helpful in clinical assessment and when looking for intraocular invasion. Magnetic resonance imaging is required to assess orbital invasion. Diffusion and perfusion-weighted MR imaging can help in differentiating invasive CSCC from other eyelid masses [70,72,73,74].

## 5. Histomorphological Features

Macroscopically CSIN lesions look as flat, cream/white epithelial thickenings/plaques. They may also be papillomatous. Invasive CSCC are gelatinous grey/white nodular, papilliform or ulcerated/erythematous masses. Lesions in the tropics can be brown and have a lot more surface keratinisation [11,35,36]. Depending on the specimen type, invasion of the cornea and other ocular structures and/or orbital soft tissue can be seen on macroscopic examination.

**Figure 3:**
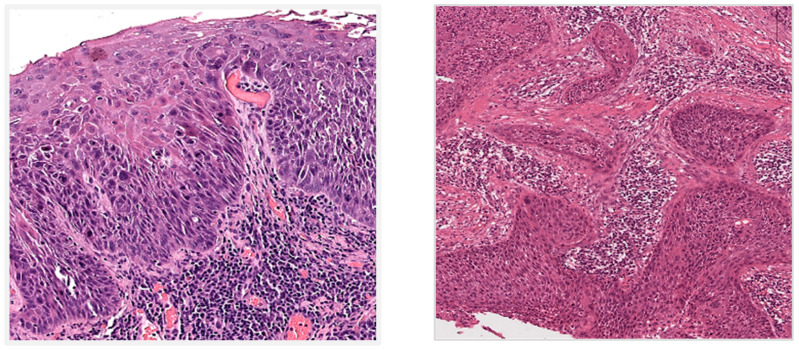
Haematoxylin and eosin staining photomicrographs showing in situ conjunctival squamous carcinoma (image on the (**left,** ×200)) and invasive CSCC (image on the (**right,** ×100)).

Microscopically CSIN lesions show a range of epithelial dysplasia (cytological atypia, architectural atypia and lack of maturation), which is graded mild (confined to lower third of epithelium), moderate (involves up to middle third), and severe (extends to upper third but surface differentiation preserved). In situ squamous carcinoma shows full-thickness epithelial dysplasia. CSIN, including in situ carcinoma, do not breach the epithelial basement membrane (Figure 3). At low magnification, an abrupt demarcation with the background normal conjunctival epithelium, and with goblet cell loss can be appreciated. At higher magnification, cytological atypia comprises nuclear enlargement and pleomorphism with increased nuclear-to-cytoplasmic ratio, hyperchromatic chromatin and prominent nucleoli. Abnormal mitotic figures are seen at varying levels of the epithelium. Dyskeratosis and apoptotic cells may also be present. Architecturally, the epithelium shows abnormal stratification with loss of normal polarity, and surface keratosis or parakeratosis. The underlying substantia propria/stroma often shows elastotic degeneration from chronic sun exposure. UV-associated cases can resemble skin actinic keratoses [35,36,75,76,77,78,79,80,81,82,83]. High-risk HPV-associated cases usually lack surface keratinisation or parakeratosis, show full thickness epithelial dysplasia and have a basaloid appearance [62,63].

Invasive CSCC usually arises from conjunctival in situ squamous carcinoma but breaches the basement membrane invading the underlying stroma (Figure 3). The invasive carcinoma can be infiltrative or exhibit pushing borders and show stromal desmoplasia. Perineural and lymphovascular invasion may be present. Histological classification of invasive CSCC includes: conventional (showing various degrees of keratinisation); basaloid (poorly differentiated with pleomorphic cells with scant basophilic cytoplasm); spindle cell (poorly differentiated with pleomorphic spindled cells); pigmented (CSCC with melanosis and melanophages); acantholytic (acantholytic and dyskeratotic cells with apoptotic debris); and with mucinous differentiation (intracytoplasmic and extracellular mucin but no true gland formation, overlying in situ component with mucinous differentiation). Immunohistochemistry is usually only required for diagnosis in poorly differentiated tumours, such as basaloid or spindle cell subtypes. For intraepithelial lesions, p53 is expressed in the dysplastic cells. In poorly differentiated invasive carcinomas, CSCC is positive for broad-spectrum/pan and high molecular weight cytokeratins (e.g., AE1/3 or MNF116 and 34βE12 or CK5/6, respectively). Diffuse nuclear and cytoplasmic ‘block-like’ p16 positivity can be predictive of high-risk HPV infection but requires molecular techniques to confirm HPV DNA or RNA presence by either polymerase chain reaction or in situ hybridisation [35,36,62,63,65,69,75,76,77,78,79,80,81,82,83,84,85,86,87,88,89,90,91].

In situ conjunctival squamous carcinoma and invasive CSSC are staged by the American Joint Committee on Cancer (AJCC) and the Union for International Cancer Control (UICC) T (tumour size), N (nodal spread), and M (metastasis) eighth edition classification system, which has been validated for the risk of tumour recurrence, development of metastasis and survival [92,93].

## 6. Treatment and Prognosis

There is no standard of care treatment and little trial evidence for the therapies in use for in situ conjunctival squamous carcinoma or invasive CSCC; consequently, management varies considerably between ophthalmic and specialised ocular oncology centres. For localised, solitary tumours, this includes surgical excision (wide local) +/− amniotic membrane allograft and +/− adjuvant cryotherapy, topical chemotherapy (mitomycin C, 5-fluorouracil or interferon alpha-2b), radiotherapy (brachytherapy, proton beam or photon external beam), enucleation, or radical orbital exenteration for advanced cases with local tissue invasion [6,12,30,31,32,94].

Surgical excision with a ‘no touch’ technique and wide margins of smaller localised tumours (<5 mm) is the most frequent approach. Alcohol application allows resection of the corneal component by corneal epitheliectomy. Adjunct cryotherapy usually applied in double- or triple-freeze–thaw cycles to the margins and base of the tumour after resection limits spread. In diffuse recurrences, for example of the tarsal conjunctiva, cryotherapy spray can be applied to limit regrowth. Eyes with intraocular invasion may be salvaged with localised radiotherapy, such as proton beam or plaque brachytherapy. If tumour control cannot be achieved, enucleation may become necessary. For advanced cases of invasive CSCC with periocular and orbital invasion, radical orbital exenteration is performed, which leads to considerable facial disfigurement [95,96,97].

Topical chemotherapy agents, such as interferon alpha 2b (IFNa-2b), 5-fluorouracil (5-FU), or mitomycin C (MMC), can be used as adjuncts to surgery or even as monotherapies in certain cases. IFNa-2b has immunomodulatory mechanisms and inhibits cell proliferation and can be administered as single subconjunctival injection or a regime of topical eye drops. MMC utilises its DNA alkylating properties to inhibit RNA synthesis and tumour growth. Antimetabolite 5-FU blocks DNA synthesis by inhibiting thymidylate sulphate [96,97,98,99,100]. In a multicentre, randomised, placebo-controlled trial, Gichuhi et al. showed that a surgical ‘no touch’ technique together with 5-FU drops four times a day for 4 weeks reduced recurrence at 1 year from 36% to 11% [101].

The HPV vaccine has been used to treat recalcitrant conjunctival in situ squamous carcinoma [102]. In this case, four intramuscular doses of a 9-valent HPV vaccine resulted in a dramatic reduction in the size of the tumour, though not full resolution. This approach has also been used in conjunctival papillomas [103,104].

Visudyne photodynamic therapy has also been tried in conjunctival in situ squamous carcinoma, though with higher rates of recurrence than excision. Its use is limited to cases that cannot have surgical excision for relatively localised disease [105]. Recently, a laser-activated anticancer virus–drug particle conjugate has been developed for small uveal melanomas (belzupacap sarotalocan or AU-011; Aura Biosciences, Boston, MA, USA). Such an approach for localised treatment of CSIN/in situ and invasive CSSC is an attractive proposition.

Radiotherapy options include brachytherapy (beta radiation using strontium-90 or ruthenium-106; gamma radiation using iodine-125) directly on the tumour or surgical bed post excision; proton bean radiotherapy (high precision delivery using Bragg peak, 48–60 Gy); gamma knife stereotactic radiosurgery; or external beam radiation. These have been used to preserve the eye, periocular tissues and ocular function; as primary treatment for unresectable invasive CSCC, patients not suitable for surgery, or post-surgery for positive/narrow margins or recurrent disease [106,107,108,109].

Postoperative complications include scarring, symblepharon formation, limbal stem cell failure, ulceration/non-healing defects, glaucoma, cataract and vision loss. Complication rates and risk of recurrence are high (5–56% recurrence; most within the first year but even after 5 years), especially in large tumours and cases with positive surgical margins, warranting close long-term follow up. Lymph node metastases occur in ~2–24% and usually involve preauricular, parotid, submandibular and/or cervical nodes, depending on invasive CSCC location. Distant metastasis may also involve the lungs, bone and spleen and brain (~6.2–18%). Risk factors for metastasis include tumour thickness, histological poor differentiation and spindle cell carcinoma subtype, and orbital invasion/high tumour staging. Poor prognostic indicators include high-staged tumours (T3/T4), positive surgical margins and nodal metastases. Overall 5-year disease-related survival in localised invasive CSCC reported to be as high as 95% but 70% in advanced (T3/T4) cases. The overall risk of tumour-related death ranges from 2 to 5% [8,32,33,34,35,36,110,111,112,113].

The use of genetics for prognostication in invasive CSCC is currently limited. However, advances in characterising invasive CSCC molecular drivers are offering insight into potential targeted therapies already in use of other cancer types. Targeted and immunotherapies have recently become promising options for advanced CSCC as an alternative to orbital exenteration although data are limited, with only those from small case series or single case studies in patients with inoperable disease or as first-line therapy prior surgery in advanced cases [37,38,39,40,41,42,43,44]. A summary of immune checkpoint inhibitors (ICIs), namely PDL-1, is presented in Table 1.

Given the overexpression of *EGFR*, its role in prognosis, and the success of EGFR inhibitors in treating head and neck SCC, El-Sawy et al. reported significant tumour shrinkage in two patients treated with EGFR inhibitors for advanced orbital SCC who had refused orbital exenteration [19,45]. Small clinical studies have investigated the use of anti-VEGF agents, such as bevacizumab and ranibizumab, given VEGF established role in angiogenesis and tumourigenesis [47,114,115]. Clinical studies on targeted therapies in CSCC patients are summarised in Table 2.

Anti-VEGF and EGFR-targeted approaches show biological activity in a subset of patients but cannot currently be considered standard of care and are best reserved for clinical trials or highly selected refractory cases.

In summary, immune checkpoint inhibitors and targeted therapies have shown very encouraging responses in advanced CSCC; however, the current evidence remains limited and of low level. All data come from small case series or isolated case reports. There are no prospective or randomised controlled CSCC-specific trials. There is clinical heterogeneity exists across cohorts (including CSCC, periocular and orbital SCC) hence limiting CSCC-specific conclusions. Response assessment (Response Evaluation Criteria in Solid Tumours/RECIST criteria) is inconsistent and not standardised, and follow-up duration is generally short, precluding robust evaluation of long-term disease control or survival benefit at present. Toxicity reporting is similarly inconsistent.

## 7. Future Direction and Conclusions

The progress in cancer genetics and immunology presents exciting new frontiers for better understanding CSCC pathogenesis. Insight into the molecular drivers of disease development and its integration with clinical and histomorphological evaluation will allow earlier diagnosis, improve risk stratification and prognostication, and identify patients for specific therapies (i.e., ‘personalised/precision medicine’). This will further enable the development of clear management guidelines and enrolment into targeted therapies earlier than current practice, facilitating improved treatment outcomes and reduce risk of metastatic disease. Other novel methods through biotechnology and bioengineering, such as biosensors and infrared biomarkers, offer exciting developments in non-invasive methods of earlier diagnosis of preinvasive disease and prognostic prediction. Innovative drug delivery systems are being developed to achieve sustained, localised drug release to minimise complication rates and improve patient compliance and treatment outcomes.

In conclusion, invasive CSCC is an ocular surface cancer with increasing global incidence. Multidisciplinary care in a specialist centre for ocular cancers is required for prompt accurate diagnosis, staging and management. Given its rarity in comparison to other SCCs, international multicentre collaboration is pivotal to obtain sufficient numbers in order to progress translational research and enlist patients into clinical trials

## Figures and Tables

**Table 1:** Anti-PDL1 therapies in invasive CSCC.

Study	Patient (s)	Stage	Primary Treatment	Agent Used	Dosage	Outcome	Adverse Reactions
Esmaeli et al., 2025 [40]	17 (5 CSCC)	Primary advanced periocular SCC (orbital, conjunctival, lacrimal)	Single systemic ICI or combined with systemic chemotherapy	Cemiplimab or Pembrolizumab	Various cycles. Dosage not specified.	Five complete responses, eight partial responses, four stable disease. At least one CSCC with nodal metastasis achieved complete response and avoided surgery	One patient diabetic ketoacidosis but others not specified
Azad et al., 2025 [41]	5	Primary advanced CSCC	Systemic ICI therapy	Cemiplimab or Pembrolizumab	350 mg IV every 3 weeks; 400 mg IV every 6 weeks, respectively	All showed progression, three required exenteration	Not specified
Kanda et al., 2025 [39]	9	Primary advanced invasive CSCC	Systemic ICI therapy	Cemiplimab	350 mg IV every 3 weeks	22% complete response, 33% partial response, 44% progressed	Hypothyroidism, hepatitis
Ceylanoglu et al., 2024 [42]	2	Primary CSIN; one patient also with metastatic cutaneous SCC	Excisional biopsy with cryotherapy	Cemiplimab	350 mg IV every 3 weeks	Response shown in metastatic lesions. CSIN continued to grow in both.	Not specified
Steren et al., 2022 [43]	11	One patient with primary CSCC with orbital involvement; 10 patients with orbital SCC	Excisional biopsy	Cemiplimab	350 mg IV every 3 weeks	Complete response in 9/11 patients	Pneumonitis, diarrhoea, fatigue, rash
Hoffmann et al., 2021 [44]	59 years old, Female	Recurrent CSCC	Radiotherapy	Cemiplimab	3 mg/kg IV every 2 weeks	Resolution after 19 months of treatment	None
Demirci et al., 2020 [37]	5	Two patients recurrent CSCC and three patients primary CSCC all with orbital involvement	Four excisional biopsy; one radiotherapy	Pembrolizumab or Cemiplimab	Pembrolizumab—350 mg every 3 weeks Cemiplimab—200 mg every 3 weeks	4/5 patients complete response; one showed progression	Colitis, rash

(Not specified = no specific details included in the study for dosage or adverse drug reactions, respectively).

**Table 2:** Targeted therapies in invasive CSSC.

Study	Patient (s)	Stage	Primary Treatment	Agent Used	Dosage	Outcome	Adverse Reactions
Asena et al., 2015 (anti-VEGF) [114]	6	Primary CSIN	Topical targeted therapy	Bevacizumab	5 mg/mL eye drops, 4 times daily for 8 weeks	2/6 complete tumour resolutions, four patients required excisional surgery	None reported
Faramarzi et al., 2013 (anti-VEGF) [115]	10	Combination of primary CSIN and CSCC	Perilesional/subconjunctival injection with excision/cryotherapy if needed	Bevacizumab	2.5 mg injection given twice 2 weeks apart	Mean tumour area reduced by 25% after first and 42% after second injection, complete resolution in two patients	None reported
Finger et al., 2012 (anti-VEGF) [47]	5	Recurrent CSCC	Excision, cryotherapy and topical interferon α or MMC	Ranibizumab	0.5 mg monthly or bimonthly	3/5 complete responses, two failed treatments	None reported
El Sawy et al., 2012 (EGFR Inhibitor) [45]	90 years old, female	Medial canthal and orbital SCC	Systemic EGFR inhibition	Erlotinib	150 mg orally once daily, increased to 200 mg orally once daily	Significant tumour reduction	None reported
El Sawy et al., 2012 (EGFR Inhibitor) [45]	81F, male	Advanced orbital SCC	Systemic EGFR inhibition	Cetuximab	400 mg/m^2^ intravenous loading dose, followed by 250 mg/m^2^ intravenous weekly	Marked tumour shrinkage	Skin reaction

## Data Availability

Not applicable.
